# Trends and cross-country disparity in the burden of pulmonary arterial hypertension among women of childbearing age from 1990 to 2021

**DOI:** 10.3389/fgwh.2025.1651601

**Published:** 2026-02-11

**Authors:** Liyuan Chen, Xiaojie Wu, Xuemei Gao

**Affiliations:** 1 Department of Obstetrics and Gynecology, Wuhan No.1 Hospital, Wuhan, China; 2 Department of Respiratory and Critical Care Medicine, Wuhan No.1 Hospital, Wuhan, China

**Keywords:** pulmonary arterial hypertension, global burden of disease study 2021, women of childbearing age, socio-demographic index, incidence, mortality, disability-adjusted life years

## Abstract

**Introduction:**

There is currently a lack of comprehensive literature analysis on the global burden and trends of pulmonary arterial hypertension (PAH) among women of childbearing age (WCBA). We fill this evidence gap by evaluating the burden and temporal trends of PAH in WCBA at global, regional, and national levels from 1990 to 2021.

**Methods:**

Data about PAH burden were extracted from the Global Burden of Disease Study (GBD) 2021. Moreover, PAH burden was explored across regions with different age, social development index (SDI) or health system.

**Results:**

There was a significant global increase in incident and prevalent cases of PAH among WCBA. Disability-adjusted life years (DALYs) and deaths initially increased but began to decline after 2010. Age-standardized incident rate (ASIR) and age-standardized prevalent rate (ASPR) increased in all SDI regions except in low SDI. Low and low-middle SDI regions bore the heaviest burden. Basic and limited healthcare systems showing the most pronounced increases in cases, but with advanced healthcare systems demonstrating a sharp reduction in age-standardized rates (ASR) of DALYs and deaths. Geographically, the highest ASIR were observed in Sub-Saharan Africa regions. Meanwhile, Central Asia and Tropical Latin America had the highest ASR of DALYs and deaths. National-level analysis identified India and China with the highest case numbers, and Sweden with the highest ASPR. Mauritius, Mongolia and Tajikistan topped ASR of DALYs and deaths.

**Conclusion:**

This study provides a comprehensive, time-series portrait of PAH burden and inequalities among WCBA worldwide. Addressing socioeconomic factors and strengthening healthcare systems are essential measures, especially in high-burden regions.

## Introduction

Pulmonary arterial hypertension (PAH) is characterized by progressive and debilitating pulmonary vascular disease, marked by a gradual increase in pulmonary vascular resistance (PVR) and a mean pulmonary artery pressure higher than 20 mmHg (1, 2). The progressive remodeling and narrowing of the pulmonary vasculature ultimately lead to right ventricular dysfunction and failure, which results in significant morbidity and mortality (3). Targeted therapies for PAH have been available over the past three decades and have significantly reduced the morbidity and mortality associated with the condition. However, these treatments are not curative, and overall outcomes remain suboptimal (1, 4). PAH is hard to diagnose and case definitions keep changing, so past studies were small and local (5–7).

The Global Burden of Disease Study (GBD) 2021 collected all available data and applied statistical and epidemiological models to establish a global estimate for the non-fatal and fatal burden of PAH (group one pulmonary hypertension) across age, sex, geographical location, and year (8). Case identification for PAH was based on clinical diagnoses confirmed via right-heart catheterization or echocardiography (9). Despite its relative rarity, PAH actually has imposed a considerable health burden worldwide, which is comparable to chronic myeloid leukemia and surpasses that of conditions like multiple sclerosis, Crohn's disease and testicular cancer (8). PAH disproportionately affects older adults and is more commonly diagnosed in females (10).

Women of childbearing age (WCBA), characterized by robust fertility and cyclical changes in sex hormones, are defined by the World Health Organization as individuals aged 15–49 years (11). While PAH affects all age groups, WCBA face unique challenges due to pregnancy-related risks. In pregnancy, extra blood can overload the stiff lung vessels of PAH patients. A reduction in PVR is typically observed by the third trimester in individuals without PAH, however, PVR remains elevated in those with PAH. This persistent elevation may exacerbate right ventricular dysfunction, ultimately leading to right ventricular failure (12). Although the highest prevalence was observed among individuals aged 75–79 years, WCBA require special attention due to the potential implications for pregnancy (8, 11). Previous international Pulmonary hypertension guidelines advised against pregnancy in women with PAH, and termination was recommended in women who do become pregnant (13). However, this stance has now shifted, reflecting accumulating evidence that pregnancy outcomes are improved in women with mild or optimally controlled PAH (1, 14). Nevertheless, pregnancy in PAH remains undeniably challenging, even in the era of targeted therapy. Counseling on appropriate contraception should be offered routinely and education for WCBA regarding potential risks is essential and should start at the time of diagnosis of PAH (14, 15). While many patients are diagnosed with PAH during pregnancy. Therefore, preconception counseling in WCBA potential with an established diagnosis of PAH is paramount (1).

Previous reports have shown global PAH epidemiology (8, 16). However, none tracked how the burden among WCBA. We fill that gap so medical staff can understand these epidemiological patterns across countries and regions (8, 16). In this study, we evaluated the number and age-standardized rates (ASR) of incidence, prevalence, disability-adjusted life years (DALYs) and mortality due to PAH among WCBA across 204 countries, 21 geographic regions, five Socio-demographic Index (SDI) regions, and four health-system tiers.

## Materials and methods

### Overview

GBD 2021 constitutes a public and systematic effort to quantify the health impacts of diseases, injuries, and risk factors across populations worldwide. This iteration of the study evaluates 371 causes of health loss within 204 countries and territories over the period from 1990 to 2021 (9). The general methodological framework for estimating disease burden in GBD 2021 builds upon established approaches documented in prior publications, incorporating updated data inputs and refined analytical techniques to enhance the accuracy and comparability of estimates (9).

#### Definition

WCBA (15–49 years) experience cyclical hormonal changes and active fertility (11).

The GBD study employs ASRs per 100,000 person-years to enable cross-population comparisons of disease burden (17).

DALYs combine years of life lost and years lived with disability, reflecting overall health burden (18).

The SDI scaled 0–1, integrates income, education, and fertility data to quantify development (19). The higher values indicate more developed regions.

The estimated annual percentage change (EAPC), quantifying the annual rate of change in ASRs, is chosen to capture long-term trends of disease burden (20).

### Data acquisition

All data on PAH in WCBA were derived from the GBD 2021. The related data of PAH burden including incidence, prevalence, deaths, DALYs, ASR, and EAPC could be freely accessible through the Global Health Data Exchange (https://ghdx.healthdata.org/gbd-2021/sources) (9).

In this study, the GBD Results Tool was employed to acquire the number and ASR for incidence, prevalence, DALYs, and mortality as measures of PAH burden from 1990 to 2021 by region, country/territory, SDI, and health system tier. Moreover, to ascertain the PAH burden across different age brackets, WCBA were categorized into seven distinct age groups: 15–19 years, 20–24 years, 25–29 years, 30–34 years, 35–39 years, 40–44 years, and 45–49 years.

### Statistical analysis

95% uncertainty intervals (UIs) were based on 1,000 iterations for each parameter and 95% UIs were defined by the 25th and 975th values of the ordered 1000 estimates. 95% UI not including zero was considered statistically significant. If the EAPC value and its 95% CI were both greater than zero, the change trend of ASR was considered upwards, and vice versa. Otherwise, the ASR was considered stable over time (21, 22). We further assessed the temporal relationship between the annual ASRs of PAH and the corresponding SDI values with Pearson's correlation in GraphPad Prism 8. When Pearson correlations were weak, a generalized additive models with spline functions were fitted to evaluate non-linear associations between SDI and age-standardized PAH burden metrics. The maps were made using ECharts software.

## Results

### At the global level

**Figure 1:**
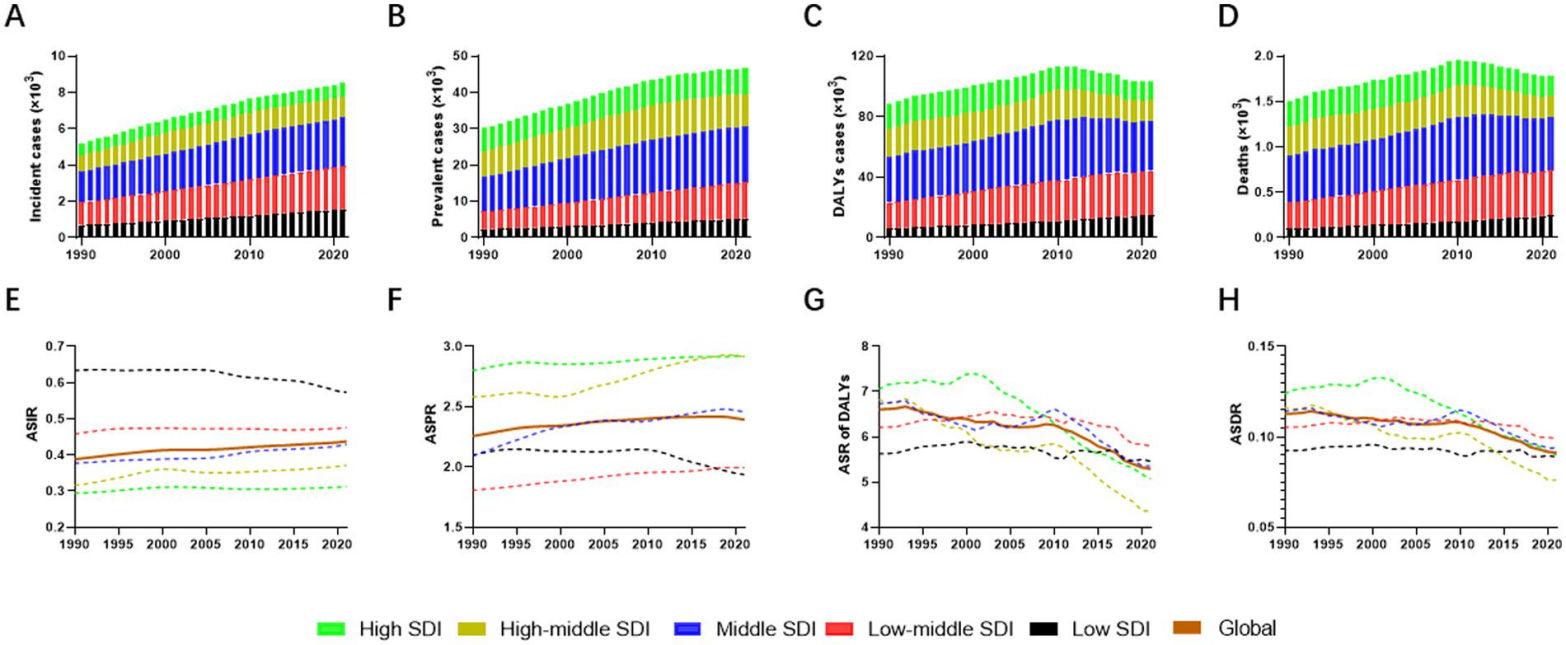
Trends of PAH burden among WCBA globally and across different SDI regions from 1990 to 2021. **(A–D)** depicted the trends of incident cases, prevalent cases, DALYs, and deaths among WCBA globally and across different SDI regions from 1990 to 2021. Incident and prevalent cases rose in all SDI regions **(A,B)**. Global DALYs and deaths increased until 2010 and declined thereafter **(C,D)**. **(E–H)** showed the age-standardized rates of PAH incidence, prevalence, DALYs, and deaths among WCBA globally and across different SDI regions from 1990 to 2021. ASIR and ASPR increased in every SDI region except low SDI **(E,F)**, whereas the ASR of DALYs and deaths fell in high- and high-middle-SDI regions **(G,H)**. PAH, pulmonary arterial hypertension; WCBA, women of childbearing age; DALYs, disability-adjusted life years; SDI, socio-demographic index.

Over the past three decades, PAH demonstrated a substantial global increase in both incident and prevalent cases among WCBA. Specifically, incident cases rose from 5,193 (95% UI: 3,821–7,051) in 1990–8,533 (95% UI: 6,304–11,497) in 2021, while prevalent cases increased from 30,186 (95% UI: 22,975–40,218) to 46,630 (95% UI: 35,395–62,267) during the same period (Tables 1, 2, Supplementary Table S1 and Figures 1A,B). DALYs add years lost to death and years lived with disability (18). The total number of DALYs and deaths showed an initial increase from 1990 to 2010, rising from 88,387 to 113,423 and from 1,507 to 1,957, respectively. However, both metrics subsequently declined to 103,151 (95% UI: 87,031–134,506) for DALYs and 1,778 (95% UI: 1,494–2,331) for deaths by 2021 (Tables 3, 4 and Figures 1C,D).

**Table 1:** The incidence of PAH among women of childbearing age in 1990/2021 and temporal trends.

Characteristics	1990		2021		1990–2021
	Incidence	ASIR/10^5^	Incidence	ASIR/10^5^	EAPC
	No × 10^3^ (95% CI)	No. (95% CI)	No × 10^3^ (95% CI)	No. (95% CI)	No. (95% CI)
Globally	5.19 (3.82–7.05)	0.39 (0.29–0.53)	8.53 (6.3–11.5)	0.44 (0.32–0.59)	0.13 (0.08–0.18)
Health system levels					
AHS	0.99 (0.73–1.35)	0.3 (0.22–0.4)	1.12 (0.81–1.53)	0.33 (0.24–0.45)	0.11 (0.05–0.16)
BHS	2.2 (1.61–3)	0.36 (0.27–0.49)	3.16 (2.3–4.27)	0.41 (0.3–0.55)	0.13 (0.04–0.23)
LHS	1.8 (1.34–2.45)	0.49 (0.37–0.67)	3.79 (2.8–5.13)	0.5 (0.37–0.68)	0.02 (−0.02–0.05)
MHS	0.2 (0.15–0.28)	0.67 (0.49–0.91)	0.45 (0.34–0.62)	0.58 (0.43–0.78)	−0.14 (−0.17–−0.11)
Socio-demographic index					
High SDI	0.67 (0.49–0.91)	0.29 (0.22–0.4)	0.76 (0.55–1.04)	0.31 (0.23–0.43)	0.06 (0.01–0.11)
High-middle SDI	0.88 (0.64–1.2)	0.32 (0.23–0.43)	1.13 (0.81–1.55)	0.37 (0.27–0.51)	0.17 (0.08–0.28)
Middle SDI	1.69 (1.25–2.3)	0.38 (0.28–0.51)	2.66 (1.94–3.61)	0.43 (0.31–0.58)	0.14 (0.05–0.23)
Low-middle SDI	1.25 (0.93–1.7)	0.46 (0.34–0.62)	2.41 (1.79–3.23)	0.48 (0.35–0.64)	0.04 (0–0.08)
Low SDI	0.71 (0.53–0.96)	0.63 (0.47–0.86)	1.57 (1.16–2.13)	0.57 (0.42–0.78)	−0.1 (−0.12–−0.07)
Region					
East Asia	1.14 (0.83–1.54)	0.34 (0.25–0.46)	1.31 (0.94–1.79)	0.4 (0.28–0.54)	0.16 (0.04–0.29)
Southeast Asia	0.46 (0.34–0.63)	0.38 (0.28–0.52)	0.8 (0.58–1.09)	0.44 (0.32–0.59)	0.15 (0.06–0.23)
Oceania	0.01 (0–0.01)	0.42 (0.31–0.58)	0.02 (0.01–0.02)	0.49 (0.36–0.67)	0.15 (0.06–0.26)
Central Asia	0.05 (0.03–0.07)	0.28 (0.21–0.39)	0.08 (0.06–0.11)	0.34 (0.25–0.46)	0.19 (0.09–0.3)
Central Europe	0.08 (0.06–0.12)	0.27 (0.2–0.38)	0.09 (0.06–0.12)	0.35 (0.25–0.48)	0.26 (0.18–0.34)
Eastern Europe	0.16 (0.12–0.22)	0.29 (0.21–0.4)	0.17 (0.12–0.23)	0.35 (0.25–0.48)	0.21 (0.12–0.3)
High-income Asia Pacific	0.12 (0.09–0.16)	0.26 (0.19–0.35)	0.11 (0.08–0.15)	0.29 (0.21–0.4)	0.15 (0.07–0.21)
Australasia	0.02 (0.01–0.02)	0.29 (0.21–0.39)	0.02 (0.02–0.03)	0.31 (0.23–0.44)	0.1 (0.02–0.2)
Western Europe	0.36 (0.26–0.49)	0.37 (0.28–0.51)	0.33 (0.25–0.46)	0.36 (0.27–0.49)	−0.04 (−0.09–0.01)
Southern Latin America	0.03 (0.02–0.05)	0.26 (0.19–0.36)	0.05 (0.03–0.07)	0.28 (0.2–0.37)	0.04 (−0.03–0.11)
High-income North America	0.17 (0.12–0.23)	0.23 (0.16–0.31)	0.21 (0.16–0.29)	0.25 (0.18–0.35)	0.12 (0.09–0.16)
Caribbean	0.03 (0.02–0.05)	0.36 (0.26–0.48)	0.05 (0.04–0.07)	0.42 (0.31–0.57)	0.17 (0.1–0.25)
Andean Latin America	0.04 (0.03–0.05)	0.41 (0.3–0.56)	0.08 (0.06–0.11)	0.46 (0.34–0.62)	0.13 (0.05–0.25)
Central Latin America	0.17 (0.13–0.23)	0.41 (0.3–0.56)	0.28 (0.21–0.38)	0.41 (0.3–0.56)	0.01 (−0.06–0.1)
Tropical Latin America	0.15 (0.11–0.2)	0.36 (0.27–0.5)	0.24 (0.18–0.33)	0.4 (0.29–0.55)	0.1 (0.02–0.2)
North Africa and Middle East	0.31 (0.23–0.43)	0.4 (0.3–0.54)	0.66 (0.48–0.89)	0.41 (0.3–0.56)	0.03 (−0.04–0.11)
South Asia	1.11 (0.82–1.5)	0.44 (0.32–0.59)	2.26 (1.67–3.03)	0.46 (0.34–0.61)	0.05 (0.01–0.09)
Central Sub-Saharan Africa	0.08 (0.06–0.11)	0.64 (0.48–0.86)	0.21 (0.16–0.28)	0.64 (0.48–0.87)	0 (−0.08–0.08)
Eastern Sub-Saharan Africa	0.34 (0.25–0.46)	0.79 (0.59–1.06)	0.79 (0.58–1.06)	0.73 (0.54–0.99)	−0.07 (−0.09–−0.04)
Southern Sub-Saharan Africa	0.09 (0.07–0.12)	0.66 (0.49–0.89)	0.14 (0.11–0.2)	0.66 (0.48–0.9)	−0.01 (−0.07–0.06)
Western Sub-Saharan Africa	0.29 (0.22–0.39)	0.67 (0.5–0.9)	0.63 (0.47–0.85)	0.52 (0.39–0.71)	−0.21 (−0.25–−0.18)

ASIR refers to the age-standardized incident rate per 100,000 population. EAPC denotes the annual percentage change. ASH represents the Advanced Health System, BSH stands for the Basic Health System, and LSH indicates the Limited Health System.

**Table 2:** The prevalence of PAH among women of childbearing age in 1990/2021 and temporal trends.

Characteristics	1990		2021		1990–2021
	Prevalence	ASPR/10^5^	Prevalence	ASPR/10^5^	EAPC
	No × 10^3^ (95% CI)	No. (95% CI)	No × 10^3^ (95% CI)	No. (95% CI)	No. (95% CI)
Globally	30.19 (22.97–40.22)	2.26 (1.72–3.01)	46.63 (35.39–62.27)	2.39 (1.82–3.2)	0.06 (0.01–0.11)
Health system levels					
AHS	9.73 (7.45–12.93)	2.92 (2.23–3.88)	10.5 (8.06–14.02)	3.07 (2.35–4.09)	0.05 (0.01–0.09)
BHS	13.11 (9.86–17.67)	2.16 (1.62–2.91)	19.45 (14.61–26.26)	2.52 (1.9–3.41)	0.17 (0.09–0.25)
LHS	6.63 (5.03–8.88)	1.82 (1.38–2.43)	15.18 (11.54–20.29)	2.01 (1.53–2.69)	0.11 (0.08–0.13)
MHS	0.69 (0.53–0.92)	2.27 (1.73–3.02)	1.46 (1.09–1.94)	1.85 (1.38–2.45)	−0.19 (−0.23–−0.15)
Socio-demographic index					
High SDI	6.35 (4.85–8.49)	2.8 (2.14–3.74)	7.09 (5.44–9.49)	2.92 (2.24–3.9)	0.04 (0.01–0.08)
High-middle SDI	7.17 (5.45–9.59)	2.58 (1.96–3.45)	8.88 (6.68–11.88)	2.91 (2.19–3.89)	0.13 (0.05–0.2)
Middle SDI	9.36 (7.09–12.58)	2.09 (1.59–2.81)	15.2 (11.44–20.52)	2.46 (1.85–3.32)	0.17 (0.1–0.24)
Low-middle SDI	4.93 (3.76–6.59)	1.81 (1.38–2.42)	10.1 (7.72–13.49)	2 (1.52–2.67)	0.1 (0.07–0.14)
Low SDI	2.34 (1.78–3.12)	2.1 (1.59–2.79)	5.31 (4–7.07)	1.94 (1.46–2.58)	−0.08 (−0.1–−0.05)
Region					
East Asia	7.06 (5.3–9.52)	2.12 (1.59–2.86)	8.61 (6.37–11.74)	2.6 (1.93–3.55)	0.23 (0.13–0.33)
Southeast Asia	2.1 (1.58–2.84)	1.75 (1.31–2.37)	3.57 (2.66–4.79)	1.95 (1.45–2.61)	0.11 (0.04–0.19)
Oceania	0.03 (0.02–0.04)	1.98 (1.49–2.66)	0.07 (0.05–0.09)	1.88 (1.39–2.52)	−0.05 (−0.14–0.03)
Central Asia	0.42 (0.31–0.56)	2.48 (1.86–3.31)	0.64 (0.49–0.85)	2.64 (2.01–3.51)	0.07 (−0.02–0.15)
Central Europe	0.84 (0.65–1.11)	2.75 (2.11–3.62)	0.72 (0.55–0.97)	2.81 (2.13–3.75)	0.02 (−0.04–0.09)
Eastern Europe	1.84 (1.41–2.45)	3.33 (2.56–4.43)	1.62 (1.24–2.16)	3.35 (2.56–4.47)	0.01 (−0.06–0.07)
High-income Asia Pacific	1.47 (1.14–1.95)	3.22 (2.48–4.27)	1.29 (0.99–1.72)	3.4 (2.61–4.51)	0.06 (0–0.11)
Australasia	0.17 (0.12–0.22)	3.08 (2.32–4.15)	0.23 (0.17–0.3)	3.12 (2.39–4.18)	0.01 (−0.07–0.09)
Western Europe	3.31 (2.53–4.39)	3.46 (2.64–4.59)	3.67 (2.83–4.9)	3.94 (3.04–5.26)	0.14 (0.1–0.18)
Southern Latin America	0.33 (0.25–0.45)	2.7 (2.06–3.6)	0.51 (0.39–0.67)	2.91 (2.23–3.87)	0.08 (0.01–0.15)
High-income North America	1.51 (1.15–2.03)	2.03 (1.55–2.73)	1.65 (1.27–2.2)	1.96 (1.51–2.62)	−0.03 (−0.07–0.01)
Caribbean	0.24 (0.18–0.32)	2.58 (1.94–3.43)	0.31 (0.23–0.41)	2.56 (1.94–3.41)	−0.01 (−0.08–0.06)
Andean Latin America	0.27 (0.2–0.36)	2.8 (2.1–3.79)	0.52 (0.39–0.69)	2.97 (2.23–3.97)	0.06 (−0.03–0.15)
Central Latin America	1.15 (0.87–1.54)	2.75 (2.08–3.67)	2.37 (1.82–3.18)	3.48 (2.67–4.66)	0.26 (0.21–0.33)
Tropical Latin America	0.97 (0.74–1.31)	2.44 (1.85–3.27)	1.7 (1.29–2.25)	2.8 (2.13–3.72)	0.15 (0.08–0.22)
North Africa and Middle East	1.67 (1.26–2.25)	2.13 (1.61–2.88)	3.64 (2.75–4.93)	2.29 (1.72–3.1)	0.07 (0–0.14)
South Asia	4.2 (3.16–5.66)	1.65 (1.24–2.22)	9.04 (6.78–12.14)	1.83 (1.37–2.46)	0.11 (0.07–0.15)
Central Sub-Saharan Africa	0.33 (0.24–0.44)	2.64 (1.98–3.55)	0.59 (0.44–0.79)	1.82 (1.35–2.42)	−0.31 (−0.38–−0.24)
Eastern Sub-Saharan Africa	1 (0.75–1.33)	2.31 (1.75–3.08)	2.14 (1.61–2.82)	1.99 (1.5–2.64)	−0.14 (−0.17–−0.1)
Southern Sub-Saharan Africa	0.24 (0.18–0.32)	1.8 (1.37–2.41)	0.44 (0.33–0.6)	2.05 (1.54–2.78)	0.13 (0.06–0.21)
Western Sub-Saharan Africa	1.04 (0.78–1.39)	2.39 (1.8–3.19)	3.3 (2.51–4.43)	2.75 (2.09–3.69)	0.15 (0.11–0.21)

ASPR refers to the age-standardized prevalent rate per 100,000 population. EAPC denotes the annual percentage change. ASH represents the Advanced Health System, BSH stands for the Basic Health System, and LSH indicates the Limited Health System.

**Table 3:** The DALYs of PAH among women of childbearing age in 1990/2021 and temporal trends.

Characteristics	1990		2021		1990–2021
	DALYs	Age standardized DALYs/10^5^	DALYs	Age standardized DALYs/10^5^	EAPC
	No × 10^3^ (95% CI)	No. (95% CI)	No × 10^3^ (95% CI)	No. (95% CI)	No. (95% CI)
Globally	88.39 (61.76–120.67)	6.61 (4.62–9.02)	103.15 (87.03–134.51)	5.29 (4.47–6.9)	−0.2 (−0.35–0.07)
Health system levels					
AHS	24.92 (21.49–26.96)	7.47 (6.44–8.08)	18.01 (16.79–19.32)	5.26 (4.9–5.64)	−0.3 (−0.37–−0.19)
BHS	41.55 (28.99–63.16)	6.84 (4.77–10.4)	41.54 (33.49–55.19)	5.39 (4.35–7.16)	−0.21 (−0.44–0.23)
LHS	20.38 (8.8–36.59)	5.59 (2.41–10.03)	39.41 (26.74–60.19)	5.22 (3.54–7.97)	−0.07 (−0.32–0.65)
MHS	1.44 (0.53–4.17)	4.74 (1.74–13.74)	4.09 (1.86–9.25)	5.18 (2.36–11.72)	0.09 (−0.22–0.66)
Socio-demographic index					
High SDI	16 (14.41–17.52)	7.06 (6.36–7.73)	12.33 (11.54–13.23)	5.07 (4.74–5.44)	−0.28 (−0.38–−0.2)
High-middle SDI	18.95 (14.82–26.15)	6.82 (5.34–9.42)	13.29 (11.08–17.5)	4.36 (3.63–5.74)	−0.36 (−0.51–−0.09)
Middle SDI	30.09 (20.05–44)	6.73 (4.48–9.84)	33.12 (26.28–41.89)	5.36 (4.25–6.77)	−0.2 (−0.42–0.26)
Low-middle SDI	16.95 (8.54–27.52)	6.21 (3.13–10.08)	29.34 (21.64–40.34)	5.79 (4.27–7.97)	−0.07 (−0.32–0.54)
Low SDI	6.29 (2.29–15.17)	5.63 (2.05–13.58)	14.98 (8.5–30.64)	5.46 (3.1–11.17)	−0.03 (−0.3–0.68)
Region					
East Asia	20.94 (12.01–35.18)	6.28 (3.6–10.55)	14.65 (9.17–23.13)	4.43 (2.77–6.99)	−0.29 (−0.62–0.46)
Southeast Asia	6.36 (3.92–11.79)	5.29 (3.26–9.81)	9.04 (6.48–15.32)	4.94 (3.53–8.36)	−0.07 (−0.35–0.64)
Oceania	0.13 (0.05–0.27)	8.09 (3.35–17.48)	0.33 (0.16–0.59)	9.55 (4.47–17.01)	0.18 (−0.25–0.91)
Central Asia	2.34 (1.51–3.1)	13.93 (9–18.49)	2.9 (1.77–4.12)	11.94 (7.29–16.97)	−0.14 (−0.41–0.21)
Central Europe	2.21 (1.88–2.55)	7.19 (6.11–8.29)	1.54 (1.36–1.75)	5.98 (5.3–6.81)	−0.17 (−0.34–0.01)
Eastern Europe	3.37 (2.72–3.91)	6.1 (4.92–7.06)	1.42 (1.23–1.64)	2.94 (2.54–3.4)	−0.52 (−0.62–−0.36)
High-income Asia Pacific	4.61 (4.34–4.91)	10.07 (9.49–10.74)	2.74 (2.52–2.94)	7.2 (6.62–7.72)	−0.29 (−0.36–−0.22)
Australasia	0.41 (0.34–0.51)	7.57 (6.26–9.47)	0.33 (0.29–0.36)	4.53 (4.06–5.04)	−0.4 (−0.55–−0.26)
Western Europe	5.76 (5.12–6.85)	6.03 (5.36–7.17)	3.88 (3.66–4.15)	4.16 (3.93–4.45)	−0.31 (−0.44–−0.21)
Southern Latin America	1.75 (1.49–2.02)	14.15 (12.02–16.32)	1.41 (1.29–1.56)	8.11 (7.39–8.97)	−0.43 (−0.53–−0.3)
High-income North America	5.46 (4.64–6.02)	7.34 (6.24–8.1)	4.96 (4.58–5.34)	5.9 (5.45–6.36)	−0.2 (−0.29–−0.08)
Caribbean	1.05 (0.73–1.47)	11.22 (7.83–15.8)	0.91 (0.59–1.46)	7.56 (4.88–12.15)	−0.33 (−0.49–−0.09)
Andean Latin America	0.6 (0.39–0.9)	6.28 (4.07–9.46)	0.71 (0.53–0.95)	4.04 (3.03–5.42)	−0.36(−0.58–0.03)
Central Latin America	1.34 (1.07–1.69)	3.2 (2.55–4.04)	1.69 (1.46–1.96)	2.49 (2.14–2.87)	−0.22 (−0.41–0.02)
Tropical Latin America	4.02 (3.75–4.26)	10.08 (9.4–10.67)	5.95 (5.55–6.31)	9.81 (9.16–10.42)	−0.03 (−0.1–0.07)
North Africa and Middle East	8.84 (5.13–13.31)	11.32 (6.57–17.03)	12.81 (7.83–17.24)	8.04 (4.91–10.82)	−0.29 (−0.58–0.26)
South Asia	13.89 (5.49–24.05)	5.45 (2.15–9.43)	26.3 (17.05–36.35)	5.32 (3.45–7.36)	−0.02 (−0.32–0.86)
Central Sub-Saharan Africa	0.4 (0.17–1.1)	3.27 (1.37–8.87)	1.15 (0.52–2.62)	3.51 (1.59–8.02)	0.07 (−0.29–0.82)
Eastern Sub-Saharan Africa	2.56 (0.91–7.87)	5.92 (2.12–18.24)	5.61 (2.41–15.46)	5.24 (2.25–14.44)	−0.12 (−0.41–0.61)
Southern Sub-Saharan Africa	0.44 (0.23–0.63)	3.28 (1.73–4.71)	0.65 (0.37–0.9)	3 (1.69–4.14)	−0.09 (−0.34–0.26)
Western Sub-Saharan Africa	1.92 (0.75–5.13)	4.4 (1.72–11.76)	4.18 (1.89–8.84)	3.49 (1.58–7.38)	−0.21 (−0.44–0.32)

DALYs refers to disability-adjusted life years. EAPC denotes the annual percentage change. ASH represents the Advanced Health System, BSH stands for the Basic Health System, and LSH indicates the Limited Health System.

**Table 4:** The death of PAH among women of childbearing age in 1990/2021 and temporal trends.

Characteristics	1990		2021		1990-2021
	Death	ASDR/10^5^	Death	ASDR/10^5^	EAPC
	No × 10^2^ (95% CI)	No. (95% CI)	No × 10^2^ (95% CI)	No. (95% CI)	No. (95% CI)
Globally	15.07 (10.36–20.75)	0.11 (0.08–0.16)	17.78 (14.94–23.31)	0.09 (0.08–0.12)	−0.19 (−0.35–0.09)
Health system levels					
AHS	4.33 (3.72–4.68)	0.13 (0.11–0.14)	3.18 (2.96–3.39)	0.09 (0.09–0.1)	−0.28 (−0.36–−0.18)
BHS	7.05 (4.84–10.89)	0.12 (0.08–0.18)	7.26 (5.75–9.82)	0.09 (0.07–0.13)	−0.19 (−0.43–0.29)
LHS	3.44 (1.43–6.22)	0.09 (0.04–0.17)	6.66 (4.49–10.34)	0.09 (0.06–0.14)	−0.07 (−0.33–0.69)
MHS	0.23 (0.08–0.71)	0.08 (0.03–0.23)	0.65 (0.29–1.5)	0.08 (0.04–0.19)	0.08 (−0.24–0.7)
Socio-demographic index					
High SDI	2.81 (2.52–3.08)	0.12 (0.11–0.14)	2.19 (2.04–2.35)	0.09 (0.08–0.1)	−0.27 (−0.37–−0.19)
High-middle SDI	3.23 (2.47–4.49)	0.12 (0.09–0.16)	2.32 (1.9–3.09)	0.08 (0.06–0.1)	−0.35 (−0.5–−0.05)
Middle SDI	5.11 (3.37–7.61)	0.11 (0.08–0.17)	5.8 (4.54–7.4)	0.09 (0.07–0.12)	−0.18 (−0.41–0.32)
Low-middle SDI	2.87 (1.41–4.67)	0.11 (0.05–0.17)	5.01 (3.7–7.07)	0.1 (0.07–0.14)	−0.06 (−0.32–0.57)
Low SDI	1.03 (0.36–2.55)	0.09 (0.03–0.23)	2.44 (1.36–5.08)	0.09 (0.05–0.19)	−0.04 (−0.31–0.72)
Region					
East Asia	3.59 (2–6.08)	0.11 (0.06–0.18)	2.6 (1.58–4.22)	0.08 (0.05–0.13)	−0.27 (−0.62–0.56)
Southeast Asia	1.07 (0.65–2.04)	0.09 (0.05–0.17)	1.56 (1.11–2.7)	0.09 (0.06–0.15)	−0.04 (−0.34–0.71)
Oceania	0.02 (0.01–0.05)	0.14 (0.06–0.29)	0.06 (0.03–0.1)	0.16 (0.07–0.29)	0.17 (−0.25–0.9)
Central Asia	0.38 (0.25–0.51)	0.23 (0.15–0.3)	0.5 (0.3–0.72)	0.21 (0.12–0.3)	−0.1 (−0.39–0.28)
Central Europe	0.39 (0.33–0.45)	0.13 (0.11–0.15)	0.28 (0.25–0.32)	0.11 (0.1–0.12)	−0.14 (−0.32–0.06)
Eastern Europe	0.56 (0.45–0.65)	0.1 (0.08–0.12)	0.24 (0.2–0.28)	0.05 (0.04–0.06)	−0.52 (−0.63–−0.35)
High-income Asia Pacific	0.81 (0.76–0.86)	0.18 (0.17–0.19)	0.49 (0.45–0.53)	0.13 (0.12–0.14)	−0.27 (−0.34–−0.2)
Australasia	0.07 (0.06–0.09)	0.13 (0.11–0.17)	0.06 (0.05–0.06)	0.08 (0.07–0.09)	−0.39 (−0.54–−0.23)
Western Europe	0.98 (0.87–1.16)	0.1 (0.09–0.12)	0.66 (0.62–0.7)	0.07 (0.07–0.08)	−0.31 (−0.44–−0.21)
Southern Latin America	0.31 (0.26–0.35)	0.25 (0.21–0.29)	0.25 (0.23–0.28)	0.14 (0.13–0.16)	−0.42 (−0.52–−0.29)
High-income North America	0.99 (0.83–1.09)	0.13 (0.11–0.15)	0.9 (0.83–0.97)	0.11 (0.1–0.12)	−0.19 (−0.28–−0.06)
Caribbean	0.18 (0.12–0.25)	0.19 (0.13–0.27)	0.16 (0.1–0.25)	0.13 (0.08–0.21)	−0.32 (−0.49–−0.08)
Andean Latin America	0.1 (0.06–0.15)	0.1 (0.07–0.16)	0.12 (0.09–0.16)	0.07 (0.05–0.09)	−0.35 (−0.59–0.04)
Central Latin America	0.22 (0.17–0.28)	0.05 (0.04–0.07)	0.27 (0.23–0.31)	0.04 (0.03–0.05)	−0.23 (−0.43–0.04)
Tropical Latin America	0.7 (0.65–0.74)	0.17 (0.16–0.18)	1.07 (1–1.13)	0.18 (0.16–0.19)	0.01 (−0.07–0.11)
North Africa and Middle East	1.48 (0.85–2.26)	0.19 (0.11–0.29)	2.21 (1.35–3.01)	0.14 (0.08–0.19)	−0.27 (−0.57–0.3)
South Asia	2.39 (0.92–4.24)	0.09 (0.04–0.17)	4.56 (2.93–6.35)	0.09 (0.06–0.13)	−0.02 (−0.32–0.93)
Central Sub-Saharan Africa	0.06 (0.02–0.19)	0.05 (0.02–0.15)	0.19 (0.08–0.44)	0.06 (0.02–0.14)	0.1 (−0.28–0.92)
Eastern Sub-Saharan Africa	0.4 (0.14–1.29)	0.09 (0.03–0.3)	0.88 (0.37–2.51)	0.08 (0.03–0.23)	−0.12 (−0.42–0.67)
Southern Sub-Saharan Africa	0.07 (0.04–0.1)	0.05 (0.03–0.08)	0.11 (0.06–0.15)	0.05 (0.03–0.07)	−0.05 (−0.32–0.35)
Western Sub-Saharan Africa	0.31 (0.11–0.86)	0.07 (0.03–0.2)	0.64 (0.27–1.42)	0.05 (0.02–0.12)	−0.24 (−0.47–0.32)

ASDR refers to the age-standardized death rate per 100,000 population. EAPC denotes the annual percentage change. ASH represents the Advanced Health System, BSH stands for the Basic Health System, and LSH indicates the Limited Health System.

### Across regions stratified by SDI

The SDI, scaled from 0 to 1, quantifies a region's sociodemographic progress, acting as a comprehensive measure that includes total fertility rate, per capita income, average education years, and age-standardized fertility rates in females under 25 (19). Based on SDI quintiles, countries were categorized into five tiers: low, low-middle, middle, high-middle, and high SDI.

A consistent upward trend in PAH incidence and prevalence was observed among WCBA across all SDI regions over the study period (Tables 1, 2, Supplementary Table S1 and Figures 1A,B). Low and low-middle SDI regions saw DALYs and deaths rise, while high and high-middle SDI regions saw them fall. (Tables 3, 4 and Figures 1C,D).

GBD employs age-standardized rates (ASRs) per 100,000 person-years to adjust for variations in age structure across populations, ensuring comparability of statistical indicators (17). Subsequently, ASRs of disease burden were evaluated. The ASIR showed a modest increase from 0.39 (95% UI: 0.29–0.53) to 0.44 (95% UI: 0.32–0.59) per 100,000 population, with an EAPC of 0.13 (0.08–0.18) (Table 1 and Figure 1E). Similarly, the ASPR increased from 2.26 (95% UI: 1.72–3.01) to 2.39 (95% UI: 1.82–3.20) per 100,000 population, with an EAPC of 0.06 (0.01–0.11) (Table 2 and Figure 1F). Notably, both ASIR and ASPR exhibited increases across all SDI regions except for the low SDI region (Tables 1, 2 and Figures 1E,F). Meanwhile, the ASR of DALYs and deaths showed subtle declines in high and high-middle SDI regions. However, these downward trends did not reach statistical significance globally, with an EAPC of −0.2 (−0.35–0.07) for ASR of DALYs and with an EAPC of −0.19 (−0.35–0.09) for ASDR (Tables 3, 4 and Figures 1G,H).

### Across healthcare system tiers

**Figure 2:**
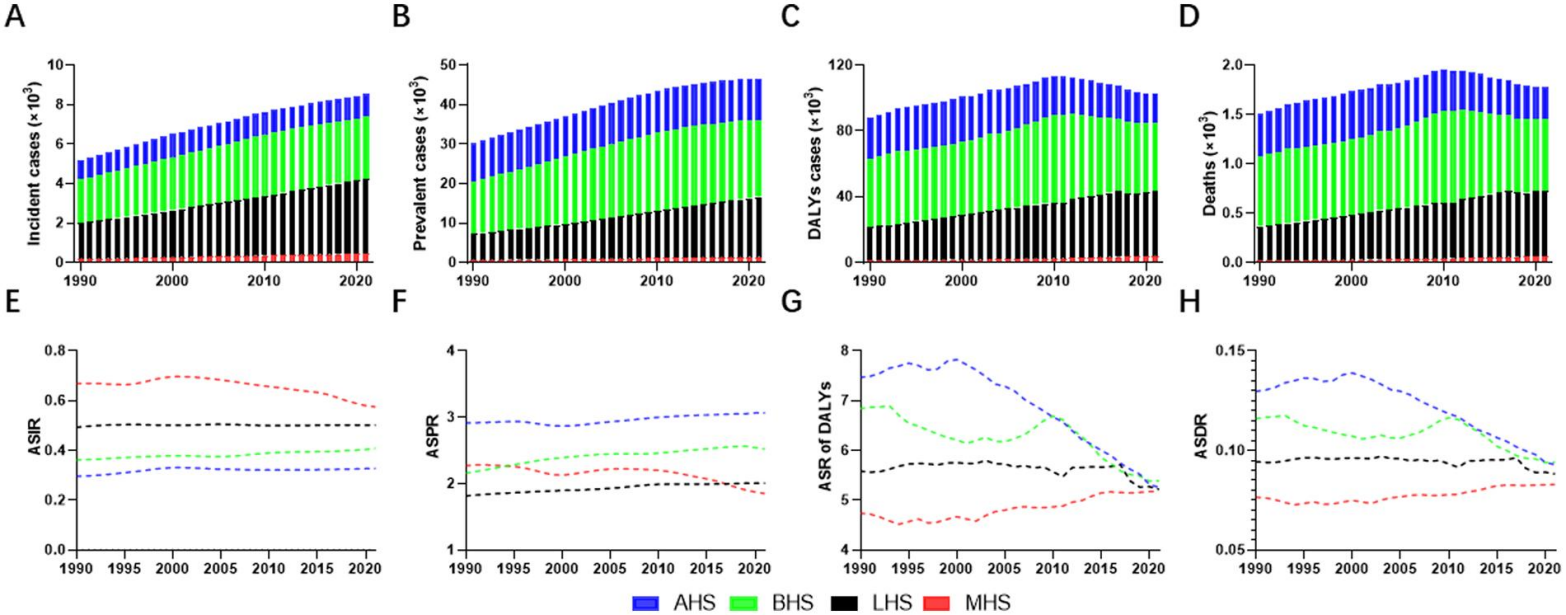
Trends of PAH burden in WCBA across different health system regions from 1990 to 2021. **(A–D)** depicted the trends of incident cases, prevalent cases, DALYs, and deaths across different health system regions among WCBA in the past 32 years, respectively. Incident and prevalent cases rose across all healthcare tiers; DALYs and deaths in BHS climbed until 2010 then fell **(A–D)**. **(E–H)** showed the age-standardized rates of PAH incidence, prevalence, DALYs, and deaths among WCBA in the past 32 years. AHS regions had seen a sharp decrease in ASR of DALYs and deaths **(G,H)**. PAH, pulmonary arterial hypertension; WCBA, women of childbearing age; DALYs, disability-adjusted life years; AHS, advanced health system; BHS, basic health system; LHS, limited health system; MHS, minimal health system.

The world was classified into Advanced Health System (ASH), Basic Health System (BSH), Limited Health System (LSH), and Minimal Health System (MSH) based on health systems tiers in GBD database (9).In addition, we conducted a comprehensive analysis of PAH epidemiology across different healthcare system contexts. The analysis revealed distinct patterns in disease burden when stratified by healthcare system levels. In terms of incident and prevalent cases, all healthcare system regions demonstrated consistent upward trends, with the most pronounced increases observed in BHS and LHS (Figures 2A,B and Supplementary Table S2). The temporal patterns of DALYs and deaths exhibited significant variation across healthcare system categories. As for DALYs and deaths, ever-increasing trends could be seen in regions with LHS and MHS throughout the 32-year study period. In contrast, the BHS regions displayed a distinct pattern, with an initial increase in DALYs and deaths during the first two decades (1990–2010), followed by a gradual decline in the subsequent decade (2011–2021). Notably, AHS regions demonstrated consistent declines in both DALYs and deaths throughout the study period (Figures 2C,D).

Analysis of age-standardized rates revealed contrasting patterns between healthcare system categories. BHS regions experienced marginal increases in ASIR and ASPR alongside substantial reductions in ASRs of DALYs and deaths. Conversely, MHS regions showed the opposite pattern. In the meantime, AHS regions had seen a sharp decrease in ASRs of DALYs and deaths. LHS regions displayed a distinct pattern, with an initial stable in DALYs and deaths from1990 to 2017, followed by substantial reductions in the subsequent four years (Figures 2E–H).

### Age-stratified analysis

**Figure 3:**
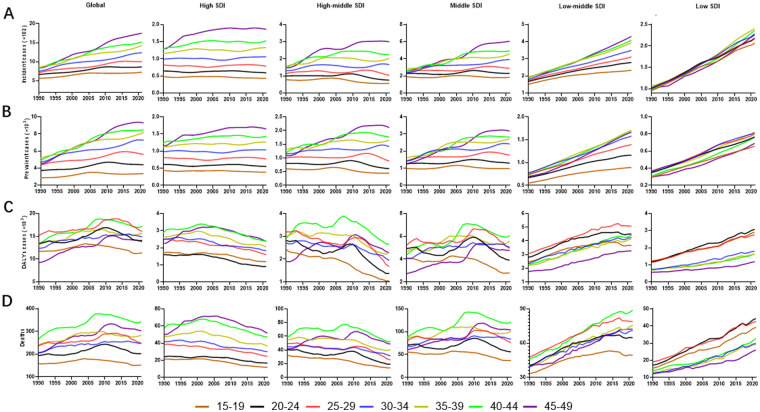
The trends of PAH burden among women of various age groups globally and across different SDI regions from 1990 to 2021. The PAH trends of incident cases **(A)**, prevalent cases **(B)**, DALYs **(C)**, and deaths **(D)** in women of various age groups were shown. Women aged 30–49 showed steady incidence/prevalence across all SDI regions, whereas only low- and low-middle-SDI areas saw significant increases among those aged 15–29 **(A,B)**. DALYs and deaths rose in every age group in low- and low-middle-SDI regions, climbing steeply with age from 15 to 19 (lowest) to 45–49 (highest) **(C,D)**. PAH, pulmonary arterial hypertension; DALYs, disability-adjusted life years; SDI, social-demographic index.

Age-stratified analysis demonstrated significant variations in disease burden patterns. Women aged 30–49 exhibited consistent in incident and prevalent cases across all SDI regions. However, cases in women aged 15–29 rose only where income is low (Figures 3A,B and Supplementary Table S3).

Temporal trends in DALYs and deaths by age group revealed distinct patterns across SDI categories. The number of DALYs and deaths increased across all age groups both in low SDI and low-middle SDI regions. In contrast, high SDI, high-middle SDI, and middle SDI regions displayed more complex trajectories of disease burden in different aged group, characterized by an initial rise followed by a decrease (Figures 3C,D). The burden of disease demonstrated a clear age-dependent pattern, with the lowest DALYs and deaths observed in the 15–19 age group and the highest burden in the 45–49 age group, showing a progressive increase with advancing age (Figures 3C,D).

### At the 21 geographic region level

At the geographic region level, our analysis revealed substantial disparities in PAH burden among WCBA from 1990 to 2021. East Asia and South Asia consistently demonstrated the highest burden, accounting for the majority of incident cases, prevalent cases, DALYs, and deaths. In stark contrast, Oceania maintained the lowest values across all metrics throughout the study period (Tables 1–4 and Supplementary Table S4).

Following age standardization, we observed pronounced regional variations in PAH burden. Four Sub-Saharan Africa regions suffered from the top four highest ASIR (Table 1). Temporal trends in ASIR showed significant regional differences. 16 regions were in upward trend of ASIR with the fastest growth in Central Europe (EAPC 0.26, 95% UI 0.18–0.34) and Eastern Europe (EAPC 0.21, 95% UI 0.12–0.30) in the past 32 years. Conversely, only two regions—Eastern Sub-Saharan Africa (EAPC −0.07, 95% UI −0.09 to −0.04) and Western Sub-Saharan Africa (EAPC −0.21, 95% UI −0.25 to −0.18)—showed significant declines in ASIR (Table 1).

Western Europe maintained the highest ASPR throughout the study period, increasing from 3.46 (95% UI 2.64–4.59) in 1990 to 3.94 (95% UI 3.04–5.26) in 2021. Central Latin America overtook Eastern Europe for second-highest ASPR by 2021 (Table 2). Notably, 11 regions experienced an increase in ASPR. But only Central Sub-Saharan Africa and Eastern Sub-Saharan Africa saw a decrease in ASPR, with an EAPC values of −0.31 (95% UI −0.38 to −0.24) and −0.14 (95% UI −0.17 to −0.10), respectively (Table 1).

In 1990, Southern Latin America, Central Asia, North Africa and Middle East and the Caribbean comprised the top four regions in terms of age-standardized DALYs. By 2021, this ranking had shifted significantly, with Central Asia, Tropical Latin America, and Oceania emerging as the regions with the highest one (Table 3). Over the past three decades, age-standardized DALYs decreased in only seven regions, with the most substantial reduction observed in Eastern Europe (EAPC −0.52, 95% UI −0.62 to −0.36). Similar patterns were observed in ASDR, mirroring the trends seen in age-standardized DALY rates (Table 3).

### At the national level

**Figure 4:**
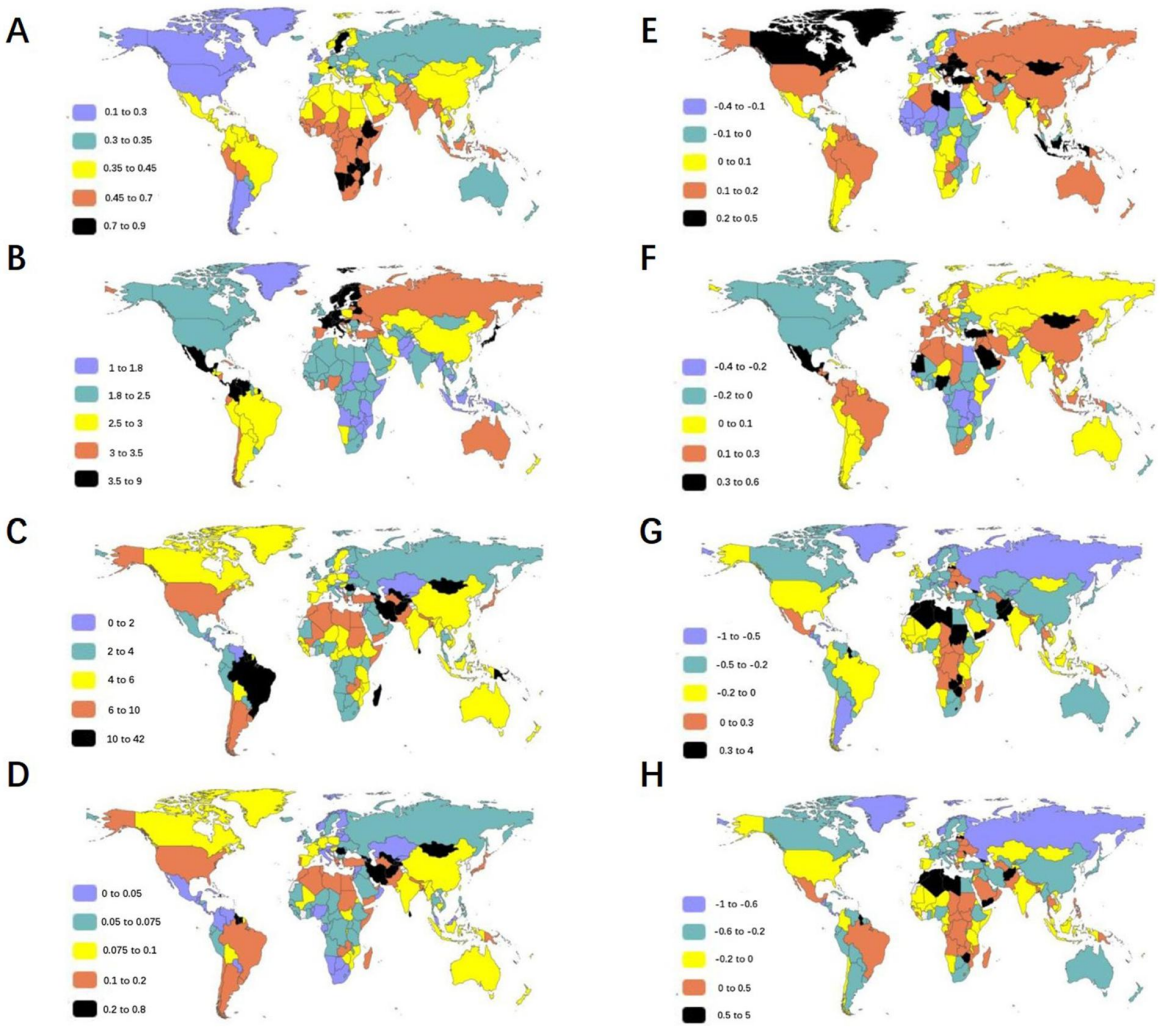
The age-standardized rates of PAH burden among WCBA in 2021 and EAPC from 1990 to 2021 in 204 countries and territories. The ASIR **(A)**, ASPR **(B)**, age-standardized rates of DALYs **(C)**, and ASDR **(D)** of PAH among WCBA around the world in 2021 were exhibited. Zambia and Botswana dominated in ASIR **(A)**, Sweden in ASPR **(B)**, while Mauritius, Mongolia and Tajikistan bore the greatest disease burden in ASR of DALYs and deaths in 2021. The EAPC of above parameters in the past 32 years were shown **(E–H)**. Slovakia and Bangladesh recorded the steepest rises in ASIR and ASPR, respectively, whereas Senegal and the Democratic Republic of the Congo showed the sharpest declines **(E,F)**. Guyana exhibited the most rapid upsurge in ASR of DALY and death, while Puerto Rico and Armenia experienced the fastest contractions **(G,H)**. PAH, pulmonary arterial hypertension; WCBA, women of childbearing age; ASIR, age-standardized incident rate; ASPR, age-standardized prevalent rate; ASDR, age-standardized death rate; DALYs, disability-adjusted life years; EAPC, estimated annual percentage change.

At the national level, our analysis revealed significant disparities in PAH burden among WCBA from 1990 to 2021.India and China were ranked as the top two countries in terms of absolute case numbers throughout this period (Supplementary Table S5). After age standardization, the burden of PAH varied greatly across different regions (Figures 4A–D and Supplementary Table S5).

The analysis of ASIR demonstrated distinct temporal and geographic patterns. In 1990, Uganda, Liberia and Senegal represented the top three countries with highest ASIR. By 2021, this ranking had shifted to Zambia, Botswana and Ethiopia (Figure 4A and Supplementary Table S5). Slovakia suffered the fastest increase in ASIR, while Senegal experienced the most substantial decline (Figure 4E and Supplementary Table S6). The examination of ASPR revealed different geographic patterns. Sweden, Netherlands and Israel had the highest ASPR in 1990. By 2021, Sweden, maintained its leading position, followed by the Netherlands and Cyprus (Figure 4B and Supplementary Table S5). Bangladesh showed the fastest ASPR increase, while Democratic Republic of the Congo demonstrated the fastest dropping (Figure 4F and Supplementary Table S6).

Analysis of age-standardized DALYs revealed evolving national patterns. In 1990, the highest burden was observed in the Bahamas, Bermuda and Barbados. By 2021, this ranking had shifted to Mauritius, Mongolia and Tajikistan (Figure 4C and Supplementary Table S5). Guyana experienced the most rapid increase of age-standardized DALYs with an EAPC of 3.72, while Puerto Rico had the lowest EAPC at −0.8 (Figure 4G and Supplementary Table S6).The pattern of ASDR mirrored that of age-standardized DALY rates (Figure 4D and Supplementary Table S5). The ASDR was highest in Bermuda, followed by Bahamas and Barbados in 1990, which were also top 3 countries in the ranking of the age-standardized DALYs in 1990 (Figure 4D and Supplementary Table S5). By 2021, the three countries with the greatest ASDR were Mauritius, Mongolia and Tajikistan (Figure 4D and Table S). As reported in Figure 4H and Supplementary Table S6, the fastest growth of ASDR was in Guyana, whereas the fastest decrease was in Armenia.

### Association with SDI

**Figure 5:**
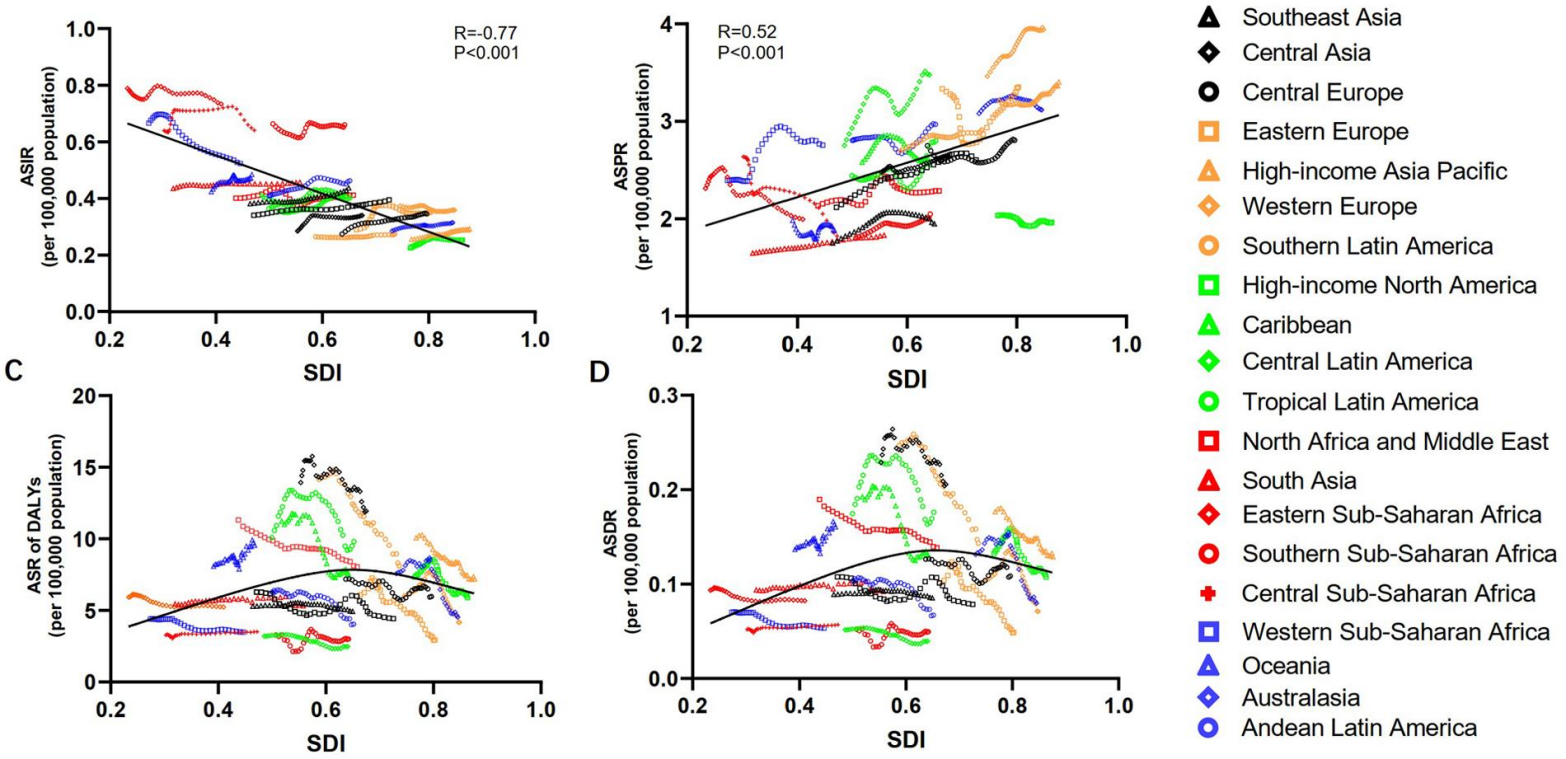
The relationship between the age-standardized rate of PAH burden among WCBA and SDI in 21 regions from 1990 to 2021. The ASIR presented negative correlation with SDI (*r* = –0.77, **(A)**). The ASPR showed positive correlation with SDI (*r* = 0.52, **(B)**). Because the Pearson correlations between SDI and both the ASR of deaths and DALYs were weak (*r* < 0.3), we employed spline-based generalized additive models to delineate their temporal trends. When the SDI was below 0.65, the age-standardized rate of DALYs and deaths exhibited a slight positive association with SDI. Conversely, when the SDI exceeded 0.65, the association became negative **(C,D)**. PAH, pulmonary arterial hypertension; WCBA, women of childbearing age; ASIR, age-standardized incident rate; ASPR, age-standardized prevalent rate; ASDR, age-standardized death rate; DALYs, disability-adjusted life years; SDI, social-demographic index.

A negative correlation was observed between ASIR and SDI across all 21 regions (*r* = −0.77, *p* < 0.001; Figure 5A). In contrast, ASPR was positively correlated with SDI (*r* = 0.52, *p* < 0.001; Figure 5B). Weak Pearson r prompted spline models for death and DALY trends. Specifically, SDI demonstrated a negative association with the ASR of DALYs and deaths when SDI values exceeded 0.65. Conversely, for SDI values below 0.65, a positive association was observed between SDI and the ASR of DALYs and deaths (Figures 5C,D). Furthermore, over the past three decades, a similar pattern of association was identified between SDI and ASR of incidence, prevalence, DALYs, and deaths among WCBA across various countries (Supplementary Figure S1).

## Discussion

### Novelty and key findings

Over the past few years, PAH has gained extensive recognition in the realms of scientific inquiry and medical clinical practice, although it has not been given adequate focus in the domains of public health and epidemiological research duo to its relative rarity and difficulty in clinical diagnosis. Women exhibit a higher susceptibility to PAH than men (23). Paradoxically, they preserve better right-ventricular function and therefore enjoy superior survival. Dysregulated estrogen synthesis and metabolism appear to be central to these sex-specific contrasts (8, 23). WCBA, typically aged 15–49, constitute a special population due to the significant threat PAH poses to maternal and neonatal health. Maternal mortality risk in PAH has been reported ranging from 17% to 50% (24, 25). Notably, maternal mortality can occur even in patients who show little or no disability before or during pregnancy. Delayed hospitalization, disease severity, and the use of general anesthesia are identified risk factors for maternal mortality (24, 25).

Recent papers covered PAH burden in all ages worldwide, but none focus on the special population of WCBA (8, 16). We provide the first full picture of PAH trends and between-country disparities for this group from 1990 to 2021. In general, the incident and prevalent cases of PAH among WCBA have surged during the past 32 years. DALYs and deaths initially increased but declined after 2010. The distribution of PAH burden across regions within the WCBA, stratified by SDI, health system, and country, exhibited significant heterogeneity. Incident and prevalent Cases rose everywhere, but deaths fell only in richer regions. Basic and limited healthcare systems had the sharpest case increases, with advanced systems showing reduced DALYs and deaths. The 45–49 age group bore the highest burden. East and South Asia reported the highest number of PAH cases, while Sub-Saharan Africa had the highest ASIR. West Europe exhibited the highest ASPR, and Central Asia along with Tropical Latin America experienced the highest ASR of DALYs and mortality. Collectively, this report provided a comprehensive understanding for epidemiological situation of PAH among WCBA, and a valuable reference for government or health-care workers in high-burden regions to combat this disease.

### Drivers of increasing cases and diverging trends

It's not difficult to discern that both incident and prevalent cases of PAH in WCBA have seen a substantial increase by a large margin worldwide and cross various regions over the study period. The substantial increase could be largely attributed to the rapid population growth, particularly in low-income areas (26). The ASIR and ASPR of PAH in WCBA exhibited slightly increases globally, which demonstrated a distinct pattern of PAH's ASIR and ASPR across all-age groups (16).The revised diagnostic criteria, such as the hemodynamic cutoff value being lowered from 25 mmHg to 20 mmHg, enlarged the enrollment patients (27, 28). It can be easily imagined that there might be plenty of undiagnostic PAH cases in 1990 owing to incomplete or unperfected standard of diagnosis. In addition, as economies and healthcare systems advance, more individuals have access to screening tests. Undoubtedly, increased screening led to a higher number of diagnosed patients.

The global five-year survival rate for PAH has seen slight improvement with the development of target medications for PAH (29). However, basic and limited healthcare systems had the sharpest case increases, with advanced systems showing reduced DALYs and deaths. Besides, our results showed the ASR of DALYs and deaths gradual declined just in high SDI region and AHS region. Treatment could only benefit the patients who was accessible to adequate medical health care. An association between SDI and different metrics of the PAH burden among WCBA were reported in our study. It could also explain why a negative association with the ASR of DALYs and deaths when SDI values exceeded 0.65. Therefore, SDI and healthcare systems had substantial impact on PAH outcomes, which were kept in line with previous publications (8, 16, 30).

With the rise in SDI, the ASPR of PAH in all-age trended upwards, whereas both the ASR of DALYs and deaths showed a decline (16)^12^. While a subtle different pattern was exhibited among WCBA. Specifically, SDI demonstrated a positive association with ASPR but a negative association with the ASR of DALYs and deaths when SDI values exceeded 0.65. Conversely, for SDI values below 0.65, a positive association was observed between SDI and the ASR of DALYs and deaths. In other words, regions with the highest ASIR and ASPR didn't coincide with those having the highest ASR for DALYs and deaths.

Regional disparities in PAH burden within WCBA were noted. Regions with high ASIR and ASPR had approximately three times the rate compared to those with low figures. Similarly, areas with high ASR for DALYs and deaths exhibited about five times the rate of those with lower value. These regional disparities may mirror the uneven distribution of risk factors associated with PAH pathogenesis. Factors such as genetic susceptibility, methamphetamine use, supplement intake, and infections like schistosomiasis or HIV differed by region and could account for the observed variations in PAH prevalence (31–33). In heritable PAH, mutations in TGF-*β* superfamily genes further derail pulmonary vascular homeostasis, driving disease initiation and progression in some regions (34). Genetic screening of high-risk individuals is therefore essential Chronic hepatosplenic schistosomiasis triggers PAH through egg-driven granulomas, sustained inflammation, vascular remodeling and fibrosis (35). Hence, intensified prevention and control of this parasitic disease offers a practical and effective route to reduce the PAH burden. To inform effective policy-making, it is crucial to grasp the epidemiological traits of PAH among WCBA in different countries. In our study, India and China were with the highest case numbers. Zambia and Sweden with the highest ASIR and ASPR, respectively. Mauritius, Mongolia and Tajikistan were ranked as top three countries in terms of ASR of DALYs and deaths. Besides, Guyana experienced the most rapid increase in ASR of DALYs and deaths. Increased focus and immediate measures are warranted in nations bearing the heaviest PAH burden among WCBA.

In nations and regions with low SDI, individuals frequently face delayed diagnosis and treatment duo to limited medical knowledge, diminished trust in healthcare and infrequent consultations. Patients do not receive timely and optical treatment with low disease control rates and poor outcome (36, 37).

With age such that the highest prevalence was among individuals aged 75–79 years (8). Our study unveiled the temporal trends in DALYs and deaths across different age groups in WCBA, revealing distinct patterns across SDI categories. These findings suggested many potential risk factors influenced on disease burden within WCBA.

### Implications for clinical practice and public health policy

Our data showed that the steepest rise in incident and prevalent cases occurred in 15–29 years old women in low- and low-middle-SDI settings. In these regions the differential diagnosis of exertional dyspnea should include PAH after anemia, asthma and congenital heart disease have been ruled out. Despite continued improvement, pregnancy in women with PAH continues to be associated with a considerable mortality risk (1, 14). Counsel at diagnosis of WCBA on pregnancy risks, offer contraception, and refer promptly to an expert pulmonary hypertension center for shared-decision counselling (1, 14). Pregnancy should be discouraged in Eisenmenger syndrome or severe, poorly controlled PAH. If it occurs, discuss termination with psychological support, or provide tertiary multidisciplinary care for those continuing (1, 14). Our findings highlight the urgent need for region-specific strategies to reduce PAH burden in WCBA, especially by tackling socioeconomic factors and strengthening healthcare in high-burden areas.

### Limitations and future directions

Several limitations have been acknowledged in this study. Firstly, the robustness and reliability of our findings primarily rely on the quality and availability of each country's vital registration system. Moreover, PAH risk factors—biological, genetic, or environmental—are unavailable in GBD 2021 beyond SDI and health-care metrics, leaving an important avenue for future investigation.

## Conclusion

The study strikingly emphasizes a substantial upsurge in the incidence and prevalence of PAH among WCBA on a global scale. the ASR of DALYs and deaths showed gradual declines in high SDI regions and AHS regions. Marked disparities are evident across diverse SDI and healthcare settings. These findings powerfully underscore the urgent and critical necessity to formulate and implement region—specific strategies aimed at alleviating the burden of PAH within WCBA. Concretely, addressing underlying socioeconomic determinants and fortifying healthcare systems are indispensable measures, particularly in regions with a high disease burden, to efficiently mitigate the impact of this debilitating disease.

## Data Availability

The original contributions presented in the study are included in the article/Supplementary Material, the datasets used in the study are available in GBD 2021.

## Supplementary material

Supplementary Figure S1 The relationship between SDI and the age-standardized rate of PAH burden among WCBA across different countries from 1990 to 2021. The ASIR presented negative associations with SDI [*r* = −0.63, **(A)**]. The ASPR showed positive associations with SDI [*r* = 0.57, **(B)**]. When the value of SDI was below 0.65, the age-standardized rate of DALYs and deaths exhibited a slight positive correlation with SDI. Conversely, when the value of SDI exceeded 0.65, the correlation became negative **(C,D)**. PAH, pulmonary artery hypertension; WCBA, women of childbearing age; ASIR, age-standardized incident rate; ASPR, age-standardized prevalent rate; ASDR, age-standardized death rate; DALYs, disability-adjusted life years; SDI, social-demographic index.
