# Supplementary figures and images for: Trends and cross-country disparity in the burden of pulmonary arterial hypertension among women of childbearing age from 1990 to 2021
Source: Front Glob Womens Health. 2026 Feb 11;6:1651601. doi: 10.3389/fgwh.2025.1651601 (PMC12932459; doi:10.3389/fgwh.2025.1651601)

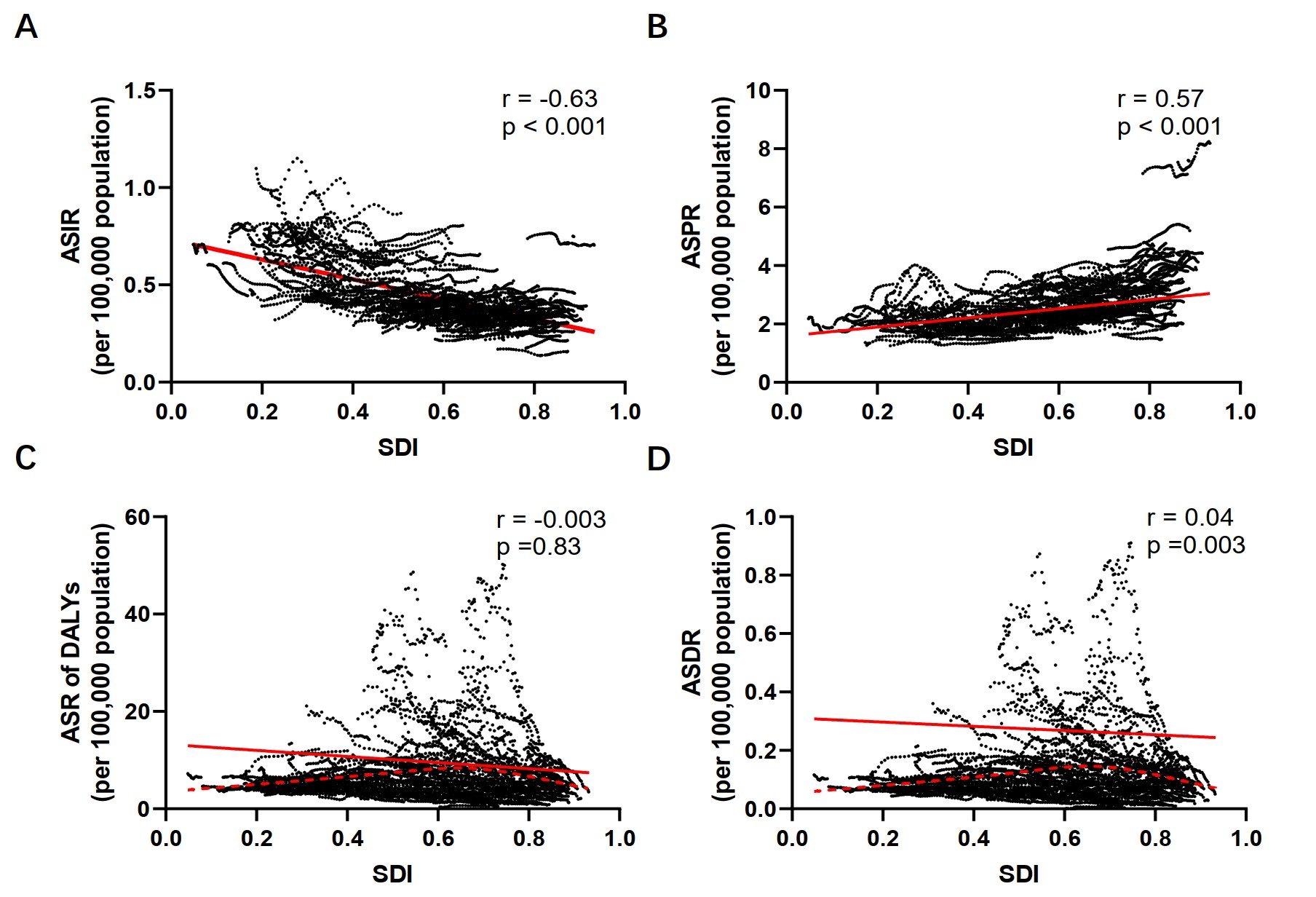

Supplement: Supplementary Figure S1 — The relationship between SDI and the age-standardized rate of PAH burden among WCBA across different countries from 1990 to 2021. The ASIR presented negative associations with SDI [r = −0.63, (A)]. The ASPR showed positive associations with SDI [r = 0.57, (B)]. When the value of SDI was below 0.65, the age-standardized rate of DALYs and deaths exhibited a slight positive correlation with SDI. Conversely, when the value of SDI exceeded 0.65, the correlation became negative (C,D). PAH, pulmonary artery hypertension; WCBA, women of childbearing age; ASIR, age-standardized incident rate; ASPR, age-standardized prevalent rate; ASDR, age-standardized death rate; DALYs, disability-adjusted life years; SDI, social-demographic index. [file Image1.jpeg]
